# Routine Histopathology in Laparoscopic Sleeve Gastrectomy Over 10 Years

**DOI:** 10.7759/cureus.69441

**Published:** 2024-09-15

**Authors:** Maaz A Yusufi, Muhammad Uneeb, Murad A Khan, Ghulam Siddiq, Muhammad Sohaib Khan

**Affiliations:** 1 Surgery, University Hospitals Dorset NHS Foundation Trust, Poole, GBR; 2 General Surgery, Shifa Tameer-E-Millat University Shifa College of Medicine, Islamabad, PAK; 3 General Surgery, Shifa International Hospital, Islamabad, PAK

**Keywords:** bariatric surgery, gastric cancer, gastrointestinal stromal tumor (gist), helicobacter pylori, histopathology, laparoscopic sleeve gastrectomy, tissue diagnosis

## Abstract

Introduction: Laparoscopic sleeve gastrectomy (LSG) is one of the world's most commonly performed types of bariatric surgery. Routine histopathology of the surgical specimen is undertaken at most institutions, regardless of the lack of clinical suspicion of any sinister pathology. We evaluated the histopathology reports of LSG cases done for morbid obesity at our hospital over 10 years to determine the distribution of gastric changes and the prevalence of *Helicobacter pylori* infection in these specimens.

Methods: A retrospective study was conducted. All LSGs performed at Shifa International Hospital, Islamabad, Pakistan, from July 1, 2014, to June 30, 2024, were assessed. A total of 538 cases were included. Histopathology reports were reviewed for all these patients, and the histopathological diagnosis and the presence or absence of *H. pylori* infection were recorded.

Results: The mean age was 36.9 ± 11.4 years. There were 201 (37.4%) males. No abnormality was found in 105 (19.5%) cases. Gastritis was present in 433 (80.5%) cases. Intestinal metaplasia was present in eight (1.5%) cases in a background of gastritis. There were no cases of gastric atrophy or dysplasia. There was one case of submucosal lipoma (0.2%) and one case of gastrointestinal stromal tumor (GIST) (0.2%). Both cases were associated with gastritis. *H. pylori* infection was present in 140 (26.0%) cases. All of these were associated with gastritis. Among the gastritis cases, 319 (73.7%) had chronic gastritis, while 114 (26.3%) had both active and chronic gastritis. The severity of gastritis, as designated by the histopathologist in their report, was minimal in 24 (4.5%) cases, mild in 228 (42.4%), moderate in 176 (32.7%), and severe in five (0.9%). There was one case of granulomatous gastritis (non-caseating) (0.2%). There was no difference in the prevalence of gastritis or *H. pylori* infection based on age or gender.

Conclusion: Histopathological examination of the LSG specimen revealed a very high prevalence of gastritis, with normal findings in less than a fifth of all cases. The prevalence of *H. pylori* infection (26.0%) is much higher in comparison to other studies. Routine histopathology of LSG specimens should continue to be performed in every case.

## Introduction

Bariatric surgery is an effective intervention for the management of morbid obesity, especially when other treatments have failed [1,2]. Laparoscopic sleeve gastrectomy (LSG) is the most commonly performed bariatric surgery in the United States of America, Canada, and the Asia/Pacific regions. Worldwide, it is the second-most-common bariatric surgery, following RYGB [3]. LSG involves resection of a part of the stomach, resulting in a surgical specimen, unlike other forms of bariatric surgery, such as gastric bypass procedures [4,5]. The pathological analysis of this surgical specimen is usually done as a routine procedure at most institutions, regardless of any clinical suspicion of any specific pathology of the stomach. However, guidelines regarding the pathological management of the surgical specimen are lacking, especially since this surgery is done for morbid obesity and metabolic syndrome rather than for any known gastric pathology.

There was a time when surgical specimens were often discarded without any further evaluation [6]. However, with the advancement of histopathological techniques, routine histopathology of all surgical specimens, regardless of any anticipated actionable findings, became common. Damjanov et al. argued against the 1926 recommendation of the Minimum Standards for Hospitals, prepared on behalf of the American College of Surgeons, which proposed “that all tissues removed at operation shall be examined in the laboratory and reports rendered thereon” [7]. Most surgical specimens now undergo some form of pathological analysis, while others are exempt. Zarbo and Nakhleh conducted a survey of 413 institutions enrolled in the College of American Pathologists in which they found that 87.1% of these institutions had written policies for determining which specimens were considered exempt from pathological evaluation and which specimens were only required to undergo gross examination [6].

Preoperative evaluation for any other gastric pathology might decrease the need for routine histopathology of the surgical specimen of LSG. However, there is a lack of consensus on the preoperative workup of patients undergoing LSG for morbid obesity, resulting in further controversy. The European Association for Endoscopic Surgery (EAES) guidelines recommend routine preoperative upper gastrointestinal endoscopy (UGIE), or radiological evaluation with a barium meal, for all patients being planned for bariatric surgery [8]. In contrast, the American Society for Metabolic & Bariatric Surgery (ASMBS) guidelines recommend a more conservative approach. They recommend that routine UGIE may be considered in all patients scheduled to undergo sleeve gastrectomy. Still, preoperative evaluation with imaging studies, upper gastrointestinal series, or UGIE should be done only in selected cases with gastrointestinal symptoms [9]. Furthermore, the incidence of gastric malignancy in Asia differs from that in Europe and the United States of America, where these guidelines were formed. The highest incidence of gastric cancer has been reported in Asia [10].

We evaluated the histopathology reports of LSG cases done at Shifa International Hospital, Islamabad, Pakistan, over the course of 10 years to determine the distribution of gastric changes and the prevalence of *Helicobacter pylori* infection in the LSG specimens in an Asian population.

## Materials and methods

Study design

A retrospective study was conducted. Approval was obtained from the Institutional Review Board and Ethics Committee at Shifa International Hospital, Islamabad (# 356-24). All LSG cases performed at SIH from July 1, 2014, to June 30, 2024, were evaluated for inclusion. Histopathology reports were reviewed for all these cases. The histopathological diagnosis and the presence or absence of *H. pylori *infection were recorded. A detailed chart review was only done for those cases where a neoplastic pathology was reported in the histopathology report. The electronic medical records (EMR) were reviewed to determine whether neoplasia had been diagnosed preoperatively. Routine preoperative UGIE or *H. pylori *testing was not done. In some cases, concurrent active and chronic gastritis were reported in the same specimen, with different severities. In such cases, a higher level of severity of gastritis was recorded. For instance, in a case with “mild active and moderate chronic gastritis,” the severity was recorded as moderate.

Patient selection

All patients who underwent LSG for obesity at our institution from July 1, 2014, to June 30, 2024, were evaluated for inclusion. Patients were included regardless of their age or gender. Both primary and revisional LSG cases were included. All LSG specimens underwent histopathological analysis and reporting at our institution.

Cases with incomplete histopathology reports were excluded. Patients who had a preoperative diagnosis of neoplasia were excluded to ensure that the surgery was of a bariatric nature. This was done through a detailed chart review of the EMR of patients with a finding of neoplasia in their LSG specimen histopathology report.

Statistical methods

Data was analyzed using IBM SPSS Statistics for Windows, Version 25 (Released 2017; IBM Corp., Armonk, New York, United States). Descriptive statistics were calculated for continuous and categorical data. Frequencies and percentages were calculated for qualitative variables such as gender and histopathological diagnosis. Mean and standard deviation were calculated for quantitative variables such as age. The distribution of histopathological diagnosis was assessed and compared among different genders and age groups to determine the effect of age and gender. A p-value less than 0.05 was considered to indicate statistical significance.

## Results

There were 543 cases of LSG performed during the study period. Four patients were excluded due to a previously known neoplastic disease, and one was excluded due to an incomplete histopathology report. Two patients underwent both primary LSG and revisional LSG, and both of their histopathology reports were included. The final number of cases included in the study was 538. The mean age was 36.9 ± 11.4 years, with a minimum age of 12 years and a maximum of 84 years (Table 1). Most cases were female, with 337 (62.6%) females and 201 (37.4%) males.

**Table 1:** Demographic details M: mean; SD: standard deviation

Parameter	N (%)
Males	201 (37.4)
Females	337 (62.6)
Age (years) (M ± SD)	36.9 ± 11.4

No abnormality was found in 105 (19.5%) cases (Table 2). Gastritis was present in 433 (80.5%) cases. Intestinal metaplasia was present in eight (1.5%) cases in a background of gastritis (Table 3). There were no cases of gastric atrophy and dysplasia. There was one case of submucosal lipoma (0.2%) and one case of gastrointestinal stromal tumor (GIST) (0.2%). *H. pylori* infection was present in 140 (26.0%) cases. All of these were associated with gastritis. Among the gastritis cases, 319 (73.7%) had chronic gastritis, while 114 (26.3%) had both active and chronic gastritis. The severity of gastritis was minimal in 24 (4.5%) cases, mild in 228 (42.4%), moderate in 176 (32.7%), and severe in five (0.9%). Among cases with both active and chronic gastritis, none had minimal severity. There was one case of granulomatous gastritis (non-caseating) (0.2%).

**Table 2:** Distribution of histopathological findings

Parameter	N (%)
Normal gastric mucosa	105 (19.5)
Minimal chronic gastritis	24 (4.5)
Mild chronic gastritis	211 (39.2)
Moderate chronic gastritis	83 (15.4)
Severe chronic gastritis	1 (0.2)
Mild chronic and active gastritis	17 (3.2)
Moderate chronic and active gastritis	93 (17.3)
Severe chronic and active gastritis	4 (0.7)

**Table 3:** Specific histopathological findings (superimposed on gastritis)

Parameter	N (%)
Granulomatous gastritis (non-caseating)	1 (0.2)
Intestinal metaplasia	8 (1.5)
Submucosal lipoma	1 (0.2)
Gastrointestinal stromal tumor	1 (0.2)

Stratification was done for age and gender. Two age groups were made, with patients younger than 45 years of age in group 1 (409 patients) and patients who were 45 or older in group 2 (129 patients). There was no difference in the prevalence of gastritis or *H. pylori* infection among the two age groups (Table 4). Similarly, there was no difference in the prevalence of gastritis or *H. pylori* infection among male and female patients (Table 5).

**Table 4:** Results stratified for age

Parameter	Group 1; N=409 (%)	Group 2; N=129 (%)	P-value
Normal mucosa	81 (19.8)	24 (18.6)	0.764
Gastritis	328 (80.2)	105 (81.4)	
*Helicobacter pylori* infection-positive	110 (26.9)	30 (23.3)	0.411
*Helicobacter pylori* infection-negative	299 (73.1)	99 (76.7)	

**Table 5:** Results stratified for gender

Parameter	Males; N=201 (%)	Females; N=337 (%)	P-value
Normal mucosa	34 (16.9)	71 (21.1)	0.240
Gastritis	167 (83.1)	266 (78.9)	
*Helicobacter pylori* infection-positive	49 (24.4)	91 (27.0)	0.502
*Helicobacter pylori* infection-negative	152 (75.6)	246 (73.0)	

## Discussion

Surgical specimens usually undergo histopathological examination and reporting, even in the absence of any definite clinical necessity or suspicion of the possibility of any actionable findings. However, some surgical specimens are often completely exempt from routine histopathological examination or might only require gross examination [7]. Specimens that are commonly exempt include placentas, prepuce following circumcision of neonates, and teeth in the absence of any gross abnormality or clinical suspicion. Most institutions have written policies or guidelines for determining which specimens must undergo only gross examination and which specimens are exempt from pathological examination [6]. There is some debate regarding adopting similar policies for other common surgical specimens, such as cholecystectomy and appendicectomy specimens [11,12]. With the rise of bariatric surgery, the number of surgical specimens following LSG has drastically risen, leading to similar concerns regarding the management of the surgical specimen [3-5].

Our study found that patients undergoing LSG had normal gastric histopathology in only a fifth of cases, with gastritis present in 80.5%. Similarly, high rates of gastritis were seen in other studies. Canil et al. found the prevalence of gastritis in LSG specimens to be 55.6%, and Almazeedi et al. found it to be 74.4% [4,13]. However, Ohanessian et al. reported that the majority of LSG specimens had no pathological changes, and gastritis was present in only 13.2% of cases [14]. They suggested this could be due to differences among pathologists regarding the definition of reporting gastritis. While some pathologists might only require the presence of lymphoid aggregates in the mucosa, other pathologists might also require significant expansion of the lamina propria with lymphoid cells to make a finding of gastritis [14]. Indeed, Pennelli et al. noted the difficulty in defining the exact number of mononucleate cells in the normal gastric mucosa [15].

There is a need for standardization of reporting these findings, not only for accurate diagnosis of gastritis but also for precise grading of its severity. Gastritis is a preneoplastic condition; therefore, precise grading of its severity is crucial. We reported the frequency of gastritis and the frequencies of different severities and types of gastritis found in our LSG specimens. We did not find any association between gastritis or *H. pylori* and age or gender. However, Safaan et al. reported an association between older age and the prevalence of intestinal metaplasia and GIST. They also reported an association between female gender and chronic active gastritis [16]. One possible explanation for this difference could be their larger sample size of 1,555 patients.

*H. pylori* infection is a known risk factor for developing gastric adenocarcinoma. A meta-analysis of 42 epidemiological studies found that *H. pylori* infection is associated with a two-fold increased risk of developing gastric adenocarcinoma [17]. We found that *H. pylori* infection was present in over a quarter of all LSG cases, all associated with gastritis. This is considerably higher than what has been reported by other authors. A review article by Nowak et al., which included 12,923 patients, found that *H. pylori* infection was present in 14.3% of cases [18]. We did not perform routine preoperative screening for *H. pylori* infection. However, most authors reported a routine practice of preoperative screening for *H. pylori* infection via UGIE and biopsy or other tests, followed by *H. pylori* eradication regimens [4,13,14,19]. However, while patients in these studies were assumed to be negative for *H. pylori* infection at the time of surgery, in all these studies, there was some positivity in the histopathology of the LSG specimen. Canil et al. reported *H. pylori* infection in 2.48% of LSG specimens, Almazeedi et al. in 7.3%, Taha-Mehlitz et al. in 5.38%, and Ohanessian et al. in 3.2% of cases [4,13,14,19]. This raises some questions regarding the accuracy of preoperative diagnostic modalities for *H. pylori* infection and the efficacy of various treatment regimens. Effective diagnosis and treatment of *H. pylori* infection is essential since it is a modifiable risk factor for developing gastric adenocarcinoma.

Premalignant changes such as gastric atrophy and intestinal metaplasia can be discovered in the histopathology of LSG specimens. We did not find any cases of gastric atrophy in any of the 538 cases included in our study. There were eight cases of intestinal metaplasia. Other authors reported similar rates of intestinal metaplasia, ranging from 0.2% to 5.14%, and gastric atrophy from 0.2% to 1.8% [4,13,14,18,19]. Spence et al. conducted a systematic review and found that both gastric atrophy and intestinal metaplasia were associated with gastric cancer; however, there was some geographical variation, with higher rates of gastric cancer associated with gastric atrophy in Asian countries [20]. We did not find any incidence of gastric cancer. However, there was one submucosal lipoma (0.2%) and one case of GIST (0.2%). Other authors have reported similar rates of GIST, ranging from 0.1% to 1.0%, as well as other neoplasms, including but not limited to gastric leiomyoma, lipoma, fundic gland polyp, gastric autonomic neural tumor (GANT), neuroendocrine tumor (NET), and gastric adenocarcinoma [4,13,14,18,19]. Overall, the incidence of neoplasia in LSG specimens was highly uncommon.

The role of preoperative UGIE and biopsy is still controversial, with different guidelines offering conflicting recommendations [8,9]. Another point to consider is that UGIE has the advantage of inspecting the entire gastric mucosa as well as taking multiple biopsies from the entire stomach. In contrast, while histopathological evaluation of the LSG specimen may be more comprehensive than UGIE biopsies, it can only assess the resected stomach but not the remnant stomach. We did not conduct routine UGIE before LSG at our center. However, all patients had histopathological evaluation of the LSG specimen. Preoperative UGIE would most likely not have significantly altered our management plan except for a few patients. However, larger, prospective studies are needed to assess this aspect of LSG, comparing the UGIE findings with the surgical specimen histopathological findings.

Some authors have recommended a more selective approach regarding the histopathology of LSG surgical specimens, considering the low number of actionable findings, especially if preoperative UGIE is done. They have cited high costs as one of the reasons for advocating for this strategy. However, routine UGIE has its own costs, often exceeding those of routine histopathology of the surgical specimen. Furthermore, the cost of histopathology is usually a tiny percentage of the total cost of the surgery. The LSG specimen histopathology costs less than 2% of the total surgery cost at our institution. Finally, the medicolegal implications also need to be considered. Histopathological evidence is extremely important in any legal proceedings arising from or following LSG, especially failure-to-diagnose claims [21]. Therefore, the cost of the histopathology seems to be more than justified.

The main limitations of this study arise from the fact that we only reviewed the histopathology reports of LSG specimens and did not perform a detailed chart review, except in those cases where neoplasia was reported. Therefore, our demographic data was limited to age and gender. Another limitation was the retrospective nature of the study. We were also unable to search for any possible associations between the patient’s risk factors for gastric malignancy and the findings on histopathology.

## Conclusions

Histopathological examination of the LSG specimen revealed normal findings in less than a fifth of all cases. The majority of cases had some form of gastritis, with variable severity. Furthermore, the prevalence of *H. pylori* infection was found to be 26.0%, which is much higher than that reported by other studies. Two cases of neoplasia were also found, one of which was GIST. Therefore, routine histopathology of LSG specimens should continue to be performed in every case, at least in the absence of preoperative UGIE. Further study is required to assess the role of UGIE and compare the findings of preoperative UGIE with LSG specimen histopathology.
